# Physicochemical and biological factors determining the patchy distribution of soil water repellency among species of dominant vegetation in loess hilly region of China

**DOI:** 10.3389/fpls.2022.908035

**Published:** 2022-10-06

**Authors:** Xiaohong Chai, Xuexuan Xu, Lushan Li, Weiwei Wang, Shuo Li, Palixiati Geming, Yuanyuan Qu, Qi Zhang, Xiuzi Ren, Yuanhui Xu, Mengyao Li

**Affiliations:** ^1^ College of Grassland Agriculture, Northwest A & F University, Yangling, China; ^2^ Institute of Soil and Water Conservation, Northwest A & F University, Yangling, China; ^3^ College of Horticulture, Gansu Agricultural University, Lanzhou, China

**Keywords:** soil water repellency, loess, plant species, hydrophobic compounds, free lipids, polar wax, microbial community

## Abstract

Soil water repellency (SWR) is a physical phenomenon whereby water cannot penetrate or has difficulty penetrating the soil surface. There are many factors involved in its occurrence, but the main factors controlling its emergence in loess remain unclear. In this work, we have studied numerous physicochemical and biological factors functioning in different dominant vegetations (*Pinus tabulaeformis* Carr., *Robinia pseudoacacia* L., and *Hippophae rhamnoides* L.) in a loess hilly region by gas chromatography–mass spectrometry (GC-MS) and high-throughput sequencing techniques. We observed that more than 75% of the soils under *Robinia* and *Hippophae* are categorized as slightly or strongly water repellent, while nearly 50% of the soils under *Pinus* are categorized as severely to extremely water repellent. The relative concentrations of total free lipids in the soil in the same water-repellency class were *Pinus* > *Robinia* > *Hippophae*, where fatty acids, alkanols, and sterols were positively correlated with SWR, whereas alkanes were not. For the abundance and diversity index of bacterial and fungal communities, the three species ranked in the following order: *Robinia* ≈ *Hippophae* > *Pinus*. Thus, solvent-extractable polar waxes were indicated to be better preserved in water-repellent soils under *Pinus* due to lower microbial diversity than *Robinia* and *Hippophae*. Here, we demonstrate polar waxes to be the principal factor controlling SWR. Moreover, the dominant phyla of fungi varied greatly than those of bacteria under three vegetation types. Correlation analysis showed that the abundance of *Actinobacteria* in dominant bacteria increased with SWR. Nonmetric multidimensional scaling suggested the fungal community in different water-repellent soils under *Pinus* to vary more than those under *Robinia* and *Hippophae*. The indicator species mainly belonged to *Actinobacteria* in bacteria and *Basidiomycota* in fungi at the phylum level; this finding was further supported by the linear discriminant analysis (LDA) effect size (LEfSe). Additionally, GC-MS identified a small amount of ergosterol, a specific biomarker of fungi under *Pinus*. These pieces of evidence collectively reveal that severe to extreme SWR occurs under *Pinus* and appears to be the most influenced by fungi and actinomycetes when the topsoil is close to air drying. However, there is a need for further testing on different plant species or land use.

## 1 Introduction

Soil water repellency (SWR) is an important transient property of soils (Hermansen et al., 2019), which occurs throughout the world. Previous studies have leaned towards considering SWR a detrimental soil property that increases surface runoff (Dekker and Ritsema, 1994), leading to more severe soil erosion and land degradation (Leighton-Boyce et al., 2007). Recently, researchers have demonstrated that some deep-rooted plants can take advantage of SWR under drought stress, resulting in the emergence of co-evolutionary behavior in natural ecosystems (Seaton et al., 2019; Smettem et al., 2021). Specifically, SWR allows rainwater to form a preferential flow, infiltrate deep soil, and store it in large quantities, making deep-rooted plants more drought resistant than shallow-rooted plants (De Boeck and Verbeeck, 2011; Zeppenfeld et al., 2017). In addition, SWR can reduce soil water evapotranspiration loss through multiple mechanisms, especially during severe drought stress, which is extremely beneficial to deep-rooted plants (Shahidzadeh-Bonn et al., 2007; Gupta et al., 2015; Rye and Smettem, 2018). Thus, deep-rooted plants are more likely to cause SWR than shallow-rooted plants. According to Alanís et al. (2016) and Lozano et al. (2013), the proportion of water-repellent soil around different tree species increases in the following sequence: trees from pine-oak forest > shrubs > shrubs and herbaceous plants > bare soil. To date, findings on SWR under different vegetation cover or land uses have been described mainly from the Netherlands, e.g., for dune sands and permanent pastures or golf greens (Doerr et al., 2005; Mao et al., 2014; Dekker et al., 2018), from Australia for farmland soils (Bond, 1972), from Germany and Spain for forest soils (Ellerbrock et al., 2005), and grassland soils in the United Kingdom (Doerr et al., 2005). However, it is still unclear how SWR is distributed in the loess of artificial forest ecosystems with different vegetation types.

The emergence of SWR can be explained as a result of interactions between water molecules and the hydrophobic compounds derived from soil organic matter/carbon (SOM/SOC) on the surface of soil particles (Mao et al., 2018). However, the relationship between SOM and SWR is still controversial. Harper et al. (2000) reported that the accumulation of sufficient amounts of SOM may induce SWR in any soil, and *vice versa;* furthermore, it was reported that the organic matter content was closely related to SWR (Mataix-Solera and Doerr, 2004; Doerr et al., 2005; Hermansen et al., 2019). Nevertheless, several studies have shown that there is no good correlation between SWR and SOM (Dekker and Ritsema, 1994; De Blas et al., 2010). Therefore, the severity of SWR is not only related to the SOC content but also to the composition of hydrophobic compounds. Up till now, different methods have been used to identify hydrophobic compounds, including n-alkanoic acids, n-alcohols, and n-alkanes (Franco et al., 2000; Horne and McIntosh, 2000; Mao et al., 2014; Mao et al., 2015), alkenoic acids, α-alkenes, terpenes, sterols (Bull et al., 2000; Franco et al., 2000; Lozano et al., 2013), and some complex biopolymers, e.g., cutin and suberin (Mao et al., 2014; Mao et al., 2015; Mao et al., 2016).

There is still uncertainty over which hydrophobic compound has the greatest responsibility for SWR. Mao et al. (2014) suggested that suberin-derived ω-hydroxy fatty acids and α, ω-dicarboxylic acids can predict SWR well. An increasing number of studies have emphasized the importance of microbially derived lipid components for SWR. For instance, microbial biomass parameters, e.g., ergosterol, and glomalin-related soil protein (GRSP), have shown to be correlated with SWR (Franco et al., 2000; Lozano et al., 2013). Epstein et al., 2010 and Schaumann et al. (2007) observed that bacteria can generate extremely hydrophobic biofilms, and most filamentous fungi secrete amphiphilic hydrophobins to form hydrophobic membranes (Wessels, 1996; Wessels, 2000; White et al., 2000; Rillig et al., 2010; Bayry et al., 2012). Additionally, the presence of the glycoprotein, glomalin, that is exuded from arbuscular mycorrhizal (AM) fungi induces SWR (Feeney et al., 2004). Fierer (2017) reviewed that the relationship between SWR and plants may not always be direct; plants may promote soil hydrophobicity through the action of their associated microbes. As a result, building a physicochemical and biological framework that explains the development of SWR by studying factors contributing to it remains a major research field (Achtenhagen et al., 2015).

Soil texture is another factor that affects SWR. Seminal work on the properties of SWR was performed on sand (Bond, 1972; King, 1981; Bisdom et al., 1993). Harper and Gilkes (1994) studied the SWR of five soil classes and considered the SWR of the soil clay content of >10% to be negligible. Sandy soils were most sensitive to high SWR, especially those with a clay content of<5% (Harper et al., 2000), where SWR decreased with clay content (Walden et al., 2015). These studies maintain that sand and clay have different sensitivities to SWR. However, if the clay forms aggregates, which reduces surface area, soils with a clay content of 25%–40% can also exhibit extreme water repellency (Crockford et al., 1991; Dekker and Ritsema, 1996). In acidic soils, SWR increases with aggregate stability (Mataix-Solera and Doerr, 2004); therefore, some research studies have reported that SWR occurs in most land-use types with permanent vegetation cover, regardless of soil texture (Doerr et al., 2006).

To date, considerable research has been conducted on SWR in sandy soils (Bull et al., 2000; Franco et al., 2000; Horne and McIntosh, 2000; Doerr et al., 2005; Morley et al., 2005; Nierop et al., 2005; Mao et al., 2014), while much less is known about the generation mechanism of SWR in loess. Loess is the most widely distributed soil on the loess plateau in China, and its particle composition is mainly fine sand (0.25–0.05 mm) and silt (0.05–0.005 mm). The SWR of loess has largely been ignored, as it is the initial soil of an eroded environment with extremely low SOC content. In recent decades, SWR of loess has emerged as an important topic, along with SOM accumulation, after the Grain for Green program (Liu and Zhan, 2019). The onset of SWR may make soil erosion more serious in loess hilly areas by increasing surface runoff, but it may also be a positive feedback effect produced by man-made ecosystems. Therefore, studying the causes of SWR in loess and clarifying the possible relationship between SWR and physical, chemical, and microbial factors after vegetation restoration have a practical significance to guide soil and water conservation in this area. In this work, we simultaneously studied the physicochemical and biological factors of three dominant vegetations in the loess hilly area. The objectives of our study are to (i) find out the differences in SWR distribution among three dominant vegetation types, (ii) compare and contrast the relative abundance and composition of solvent-extractable lipids and microbial communities in the different water-repellent soils, and (iii) investigate the key mechanisms affecting the distribution of different SWR in loess under these vegetation types. Our working hypotheses are that (i) evergreen trees (e.g., *Pinus*) provide more SOM and hydrophobic compounds than do deciduous plants (e.g., *Robinia* and *Hippophae*), leading to a more severe SWR, (ii) plant-derived compounds are better preserved in water-repellent soils under *Pinus* due to lower microbial diversity than under *Robinia* and *Hippophae*, and (iii) the distribution of SWR may be governed by different mechanisms under these three species, where solvent-extractable polar waxes may be the most relevant factor. In addition, SWR under *Pinus* seems to be the most influenced by fungi and actinomycetes. The findings from this research can contribute toward a better understanding of the role of SWR in loess under artificially reconstructed forests.

## 2 Materials and methods

### 2.1 Description of experimental sites

The sites chosen for this work are located in Changwu Agricultural Ecology Experimental Station of the Chinese Academy of Sciences (35°12′ N, 107°40′ E; 1,200 m a.s.l.), China. The average annual precipitation is 584 mm, which predominantly falls from July to August. The annual average temperature is 9.1°C, and the average frost-free period is 171 days (Li et al., 2020). The region is characterized as a semi-humid continental monsoon climate in the warm temperate zone. The soil is Malan loess. See **Supplementary Table S1** for soil texture. In the research area, *Pinus tabulaeformis* Carr., *Robinia pseudoacacia* L., and *Hippophae rhamnoides* L. woodlands were selected based on the same aspect (south slope), slope (5°–15°), slope position (middle slope), and elevation (1,100 m). The basic conditions of the three woodlands are as follows. (i) For *Pinus* forest (107°38′ N, 35°10′ E), the average tree age was 30 a, tree spacing was 2.5 m, canopy density was 85%, the thickness of the fallen leaves was 3–4 cm, and there was very little grass [dominated by *Bothriochloa ischaemum* (L.) Keng] growing under the trees. (ii) For *Robinia* forest (107°40′ N, 35°12′ E), the average tree age was 14 a, tree spacing was 1.5–3.0 m, canopy density was 70%, and the thickness of the fallen leaves was −1 cm. There were some grasses (dominated by *Festuca elata* Keng ex E. Alexeev, Astragalus sinicus L., and *Artemisia lavandulaefolia* DC., etc.) growing under the trees. (iii) For *Hippophae* forest (107°42′N, 35°15′E), the average tree age was 30 a, tree spacing was 2.0 m, canopy density was 50%, the thickness of the fallen leaves was 1–2 cm, and there were some grasses [dominated by *Agropyron cristatum* (L.) and *Gaertn.*, *Coronilla varia* L., etc.] growing under the trees.

### 2.2 Soil sampling

Samples were collected in May 2021 when the topsoil was nearly air-dried. Three 5 × 5 m square sampling plots with a 10-m distance between the plots were established under each vegetation type, and 50 sampling points were arranged in each sampling plots, where each sampling point was ~50 cm away from a tree trunk. Before sampling, the water droplet penetration time test (WDPT) (Letey, 1969; Doerr, 1998) was performed to measure the SWR of the topsoil at each sampling site (see *Section 2.3* for details). The relative frequency of the occurrence of each water-repellency class was calculated. The water-repellency classes were divided into the following five grades: wettable (WDPT< 5 s), slightly water repellent (5 s< WDPT< 60 s), strongly water repellent (60 s< WDPT< 600 s), severely water repellent (600 s< WDPT< 3,600 s), and extremely water repellent (WDPT > 3,600 s) (Bisdom et al., 1993). After the WDPT measurement at each sampling point, soil samples were collected from the first 3 cm of the topsoil depending on the water-repellency classes. At the same sampling plots, samples belonging to the same water-repellency class were blended to form a mixed sample. A total of 15, 6, and 9 soil samples were thus collected from under *Pinus*, *Robinia*, and *Hippophae*, respectively. Each mixed sample was divided into two parts, sealed in pre-sterilized non-enzyme centrifuge tubes, stored on ice, and brought back to the laboratory for analysis. One part was air-dried for the determination of soil physicochemical properties, potential SWR, and free lipids. The remaining part was stored at -80°C for DNA extraction. To distinguish the samples well, the vegetation types of *Pinus*, *Robinia*, and *Hippophae* were numbered T_1_, T_2_, and T_3_, respectively. The samples were also numbered according to the numbers of vegetation types, and the first letter of their water-repellency class, e.g., the slightly water-repellent samples under *Pinus* were numbered T_1__S_1_ and the strongly water-repellent samples under *Pinus* were numbered T_1__S_2_. In particular, the wettable soil under *Pinus* was almost bare, i.e., with little to no litter coverage.

### 2.3 Determination of *in situ* SWR

Before the WDPT test, plants and litter were removed from the soil surface, and a wireframe (100×50 cm^2^, containing 50 small squares of 10×10 cm^2^) was placed on the cleared spot. Three drops of distilled water (approximately 0.05 ml per drop) were then dropped into a small square in sequence. The times required for the complete penetration of the water droplet were recorded. The average time for triplicate drops to penetrate was taken as the WDPT value of a sample. Penetration times were classified in water-repellency classes outlined by Bisdom et al. (1993).

### 2.4 Laboratory methods

Unrefrigerated soil samples were dried to constant weight at room temperature (20°C–25°C) and sieved (2 mm) to remove coarse soil particles before analysis. Soil pH was measured by the potentiometric method (Metson, 1956) using a pH meter (PHSJ-4F, INESA Co., Ltd., Shanghai, China). For this, deionized water was used to leach the solution (1:2.5 w:v) at 25°C (Lozano et al., 2013). The SOC content was determined by the potassium dichromate-sulfuric acid external heating method (Walkley and Black, 1934), and soil available nutrient content was determined using standard methods.

To measure the potential SWR, ~15 g of soil was placed in a 50-mm-diameter aluminum box and exposed to controlled laboratory conditions (20°C, ~50% relative humidity) for 1 week to eliminate potential impacts of preceding atmospheric humidity on SWR. The potential SWR was determined using the WDPT test (Letey, 1969; Doerr, 1998) used for *in situ* SWR measurements.

To extract free lipids from the soil, according to the method of Mao et al. (2014), 30 g of soil was weighed and placed in a Soxhlet extractor (AI-ZFCDY-6Z, Na ai Co., Ltd., Shanghai, China). DCM/MeOH (9:1 v:v) was used as a solvent to obtain the extract at 70°C for 24 h (Lozano et al., 2013). The solvent was removed with a rotary evaporator (R-215, BUCHI Lab. AG, Flawil, Switzerland). After redissolving the lipids in the solvent, the extracts were passed through an SPE column filled with anhydrous Na_2_SO_4_ (2000 mg, 6 ml) to remove residual water and were dried using a gentle stream of nitrogen. Before analysis, the extracts were methylated using 500 µl of toluene, 100 µl of methanol, and 100 µl of (trimethylsilyl)diazomethane (TMS-CH_2_N_2_) at room temperature. The extracts were then eluted over a small silica gel (100–200 mesh) column with ethyl acetate and were silylated using N,O-bis(trimethylsilyl)trifluoroacetamide (BSTFA) in pyridine at 70°C for 30 min.

Extracts were analyzed using a triple quadrupole gas chromatography–mass spectrometry (GC-MS) instrument (GCMS-TQ8050NX, Shimadzu Production Co., Kyoto, Japan) with a mass range of *m/z* 50–800. One microliter of the derivatized extracts was injected onto an SH-Rxi-5Sil MS capillary column (Shimadzu 30 m × 0.25 mm inner diameter × 0.25 μm film thickness) using helium as the carrier gas at a constant flowrate (1.0 ml·min^−1^) and pressure (100 kPa). The oven heating program was followed as per the methods of Mao et al. (2014). Based on GC-MS analysis, the relative response factors of compound groups, e.g., alkanes, alcohols, and fatty acids, were so similar that they could barely be discriminated between the various types of compounds. A known amount of squalene was added to the extract as an internal standard. Compounds were identified against mass spectra from NIST libraries, interpreted spectra, retention times, or comparison to literature data. Compounds were quantified by GC-MS chromatographic peak area integration while using the following formula to correct for possible co-eluting compounds.


$$m_{i}=A_{i}\;/A_{is}\times m_{is}\times {\text{f}}_{{\text{i}}^{\prime }}\times \text{α}$$

where m_i_ is the quality of the object to be measured, A_i_ is the peak area of the object to be measured, A_is_ is the peak area of the internal standard, M_is_ is the quality of internal standard, F_i′_ is the relative correction factor, and α is the conversion coefficient.

Next-generation sequencing was employed to characterize the soil microbiome. Before extraction of total soil DNA, T_1_ and T_3_ soil samples belonging to the slightly and strongly water-repellency classes, respectively, were blended to form mixed soil samples “T_1__SS” and “T_3__SS” in a sterile environment. The soil samples belonging to the severely and extremely water-repellency classes were similarly treated to form a mixed sample “T_1__SE.” The other samples were not blended. Soil DNA was extracted from approximately 0.5 g of soil per sample using an E.Z.N.A.^®^ Mag-Bind Soil DNA Kit (M5635-02, Omega Bio-tek, Inc., Norcross, GA, USA) according to the manufacturer’s instructions. The DNA samples were diluted to 20 ng·μl^−1^ before PCR amplification. The PCR products were run on the electrophoresis gel together with the negative PCR control to verify that they were not contaminated. Each sample was treated in triplicates. The hypervariable regions, V3–V4, of bacterial 16S rRNA genes were amplified using the barcode primers: 338F (5′-ACTCCTACGGGAGGCAGCAG-3′) and 806R (5′-GGACTACHVGGGTWTCTAAT-3′). The fungal ITS1 region was amplified using ITS1 (5′- CTTGGTCATTTAGAGGAAGTAA-3′) and ITS2 (5′-GCTGCGTTCTTCATCGATGC-3′) (Schoch et al., 2012; Young et al., 2012; Sun et al., 2016; Jiang et al., 2018). The PCR products were sent to Shanghai Personal Biotechnology Co., Ltd. (Shanghai, China) for high-throughput, paired-end sequencing on the Illumina NovaSeq PE250 platform.

The sequences were then de-multiplexed, filtered, denoised, merged, quality-checked, and freed from chimeric sequences using a combination of QIIME2 (https://qiime2.org) (Bolyen et al., 2019) with DADA2 methodology (Callahan et al., 2016) and VSEARCH (Rognes et al., 2016). The processed reads were assembled into amplicon sequence variants (ASVs). After obtaining the ASV representative sequence, statistics were compiled on length distributions to remove the sequence with abnormal lengths. Filtered sequences of bacteria and fungi were matched using the GreenGenes (DeSantis et al., 2006) and UNITE (Kõljalg et al., 2013) databases, respectively. Taxonomy was assigned to ASVs using QIIME2 with classify-sklearn methodology (Bokulich et al., 2018). Singletons and ASVs appearing in only one sample were removed from ASV tables following the taxonomic assignment. All non-bacterial and non-fungal ASVs were removed. The microbial community data were flattened to achieve a minimum number of sequences. The alpha diversity index of soil bacterial and fungal communities was estimated by calculating ASV richness using QIIME2. Dilution curves were drawn using the “vegan” package in R 4.1.2, and nonmetric multidimensional scaling (NMDS) ordinations were generated using the “vegan” package in R based on Bray–Curtis dissimilarities to estimate the beta diversity in soil microbial communities (Liu et al., 2020). The sequence raw datasets in this research were deposited in the National Center for Biotechnology Information (NCBI) Sequence Read Archive (SRA) (https://trace.ncbi.nlm.nih.gov/Traces/sra/) under the accession numbers PRJNA823826 (bacteria) and PRJNA820436 (fungi).

### 2.5 Statistical analysis

To analyze the SWR performances of the different vegetation types more intuitively, Surfer 18 was used to draw the contour map of different water-repellency classes. All data were tested for normality and homogeneity of variance using IBM SPSS Statistics 26. Data were log-transformed when necessary, and one-way ANOVA was performed using the least significant difference (LSD) method, where the significance level was set to 0.05 to evaluate the significance of differences in soil physicochemical factors across the different water-repellency classes beneath different vegetations. Pearson’s or Spearman’s correlation coefficient (r) was calculated to quantify the relationship between the parameters. Linear discriminant analysis (LDA) effect size (LefSe) was performed to investigate the differences in the bacterial and fungal relative abundances across all groups with the Genescloud statistical package.

## 3 Results

### 3.1 *In-situ* SWR and potential SWR

**Figure 1:**
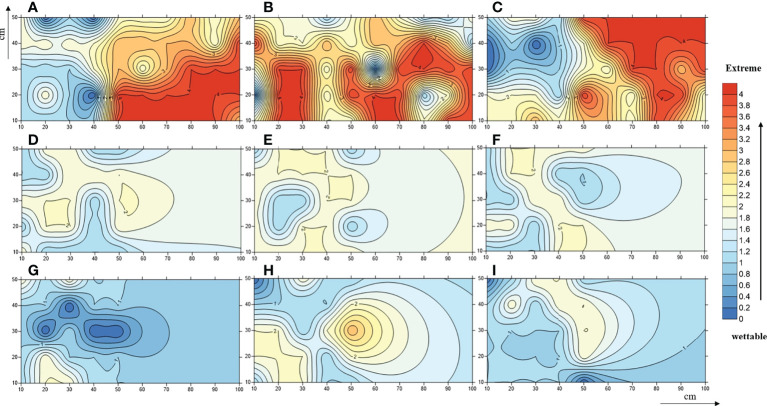
A contour map of soil water-repellence classes beneath *Pinus* **(A–C)**, *Robinia* **(D–F)**, and *Hippophae* **(G–I)**. Numbers 0, 1, 2, 3, and 4 represent wettable, slightly water-repellent, strongly water-repellent, severely water-repellent, and extremely water-repellent soils, respectively.

**Figure 2:**
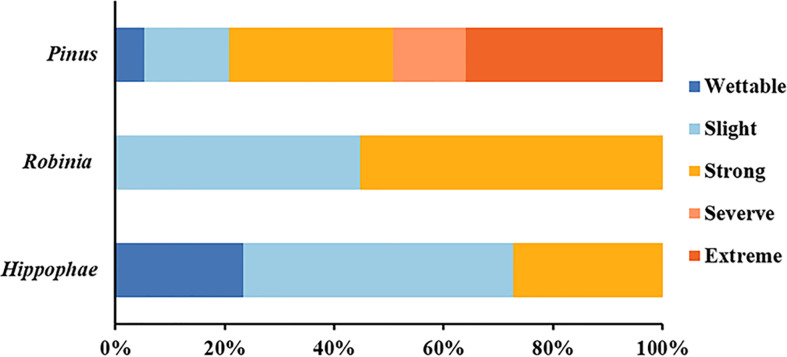
Relative frequency of occurrence of each water-repellency class beneath the different plant species (n = 450).

*In-situ* measurements showed that the SWR under the three plant species was distributed in patches in the loess hilly region (**Figure 1**). Overall, nearly 90.44% of the tested soils accounted for water repellency, with the majority classified as slightly (36.44%) to strongly (37.56%) water repellent (**Supplementary Table S2**). Severely to extremely water-repellent soils were only observed under *Pinus* (T_1_). Although all soils found under *Robinia* (T_2_) were water repellent, the strongest SWR was found under T_1_ (**Figure 2**), indicating that SWR varies with plant species (Lozano et al., 2013).

In addition to extremely water-repellent samples (T_1__E), WDPT values were reduced by repeated measurements in the laboratory (**Table 1**), and for some samples, such as T_1__S_2_, T_2__S_2_, and T_3__S_2_, water-repellency classes were altered accordingly. Thus, SWR is not a static soil property (Doerr and Thomas, 2000), and its instability is also prominent.

**Table 1:** Comparison of actual *in-situ* SWR and potential SWR.

Sample plot	Treatments	Actual *in situ* SWR(s)	Potential SWR(s)
*Pinus*(T_1_)	T_1__W	2.29	1.00
	T_1__S_1_	26.65	9.60
	T_1__S_2_	274.75	28.33
	T_1__S3	1079.51	789.70
	T_1__E	>3600	>3600
*Robinia*(T_2_)	T_2__S_1_	37.54	9.30
	T_2__S_2_	115.58	31.00
*Hippophae*(T_3_)	T_3__W	2.72	1.00
	T_3__S_1_	29.61	7.00
	T_3__S_2_	129.76	38.00

### 3.2 Physicochemical factors

#### 3.2.1 Soil pH and SOC

**Figure 3:**
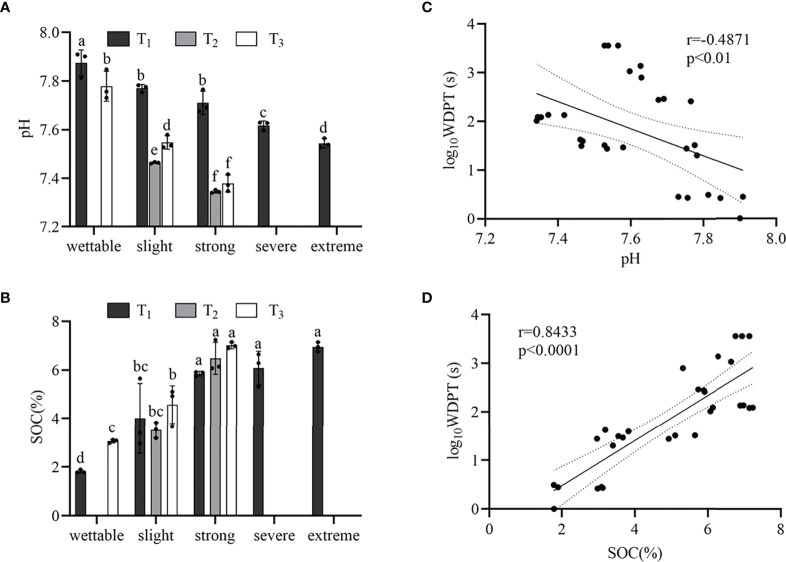
pH **(A)** and SOM content **(B)** beneath the different sample plots. Relationships between SWR (log_10_WDPT) and pH **(C)** or SOC content **(D)**. Standard errors in bars. Different letters show the statistically significant differences between the different plant species (*p*< 0.05, n=3).

The pH of wettable soil varied from 7.78 to 7.87, and that of hydrophobic soil varied from 7.35 to 7.77 (**Supplementary Table S3**). **Figure 3A** shows that the pH decreased with SWR (except for T_1__S_1_ and T_1__S_2_) under the same plot. Moreover, Pearson’s correlation was significant but weak between SWR and pH (r = −0.4871, *p*< 0.01) (**Figure 3C**).

From wettable to strongly water-repellent soil, the SWR gradually increased with SOC (**Figure 3B**), and there was a strong linear correlation (r = 0.8433, *p<* 0.0001) (**Figure 3D**). However, from strongly to extremely water-repellent classes under T_1_, there was no significant difference in SOC. In addition, we observed different water-repellency classes despite having similar SOM under the different vegetation types.

#### 3.2.2 Available nutrients in the soil

Available nitrogen (AN) and available potassium (AK) were related to SWR, but available phosphorus (AP) exhibited no correlation with SWR (**Supplementary Table S5**). In the same water-repellent class, the change in available nutrients depended on the vegetation type (**Table 2**).

**Table 2:** Available nutrient content in the soil beneath the different tree species.

Sample plots	Treatments	Available nitrogen (AN, mg·kg^−1^)	Available phosphorus (AP, mg·kg^−1^)	Available potassium (AK, mg·kg^−1^)
T_1_	T_1__W	74.73 ± 2.755de	13.59 ± 1.332g	166.56 ± 3.008c
	T_1__S_1_	83.47 ± 7.847cd	16.17 ± 0.777f	168.71 ± 4.555c
	T_1__S_2_	123.72 ± 5.893a	19.33 ± 0.541e	214.09 ± 21.381b
	T_1__S3	102.29 ± 10.388b	12.75 ± 2.252g	207.97 ± 18.365b
	T_1__E	97.18 ± 2.37b	30.09 ± 1.276c	246.09 ± 4.234ab
T_2_	T_2__S_1_	71.98 ± 2.763de	34.83 ± 1.332b	256.73 ± 34.229a
	T_2__S_2_	67.81 ± 1.892ef	41.49 ± 0.917a	230.09 ± 5.307ab
T_3_	T_3__W	55.43 ± 9.012gf	35.00 ± 0.350b	177.89 ± 13.808c
	T_3__S_1_	67.76 ± 9.049ef	36.74 ± 0.803b	217.79 ± 16.688b
	T_3__S_2_	90.21 ± 2.995bc	25.12 ± 0.095d	227.57 ± 3.689ab

Values are means ± SE (n = 3). Different letters show statistically significant differences (p< 0.05) between the different samples by ANOVA. The number of soil samples is 30.

### 3.3 Free lipids in soil

#### 3.3.1 Fatty acids

**Figure 4:**
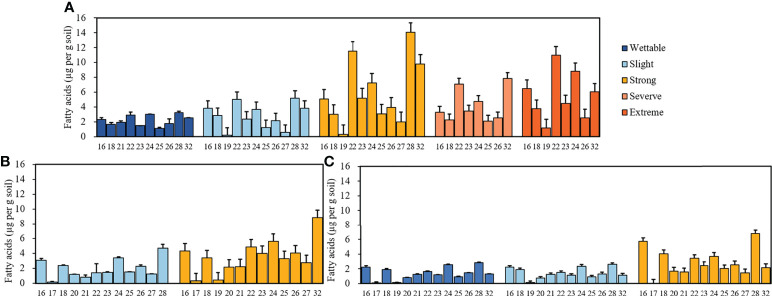
Relative concentrations of fatty acids with different carbon numbers beneath *Pinus* **(A)**, *Robinia* **(B)**, and *Hippophae* **(C)** (n=3).

**Supplementary Figure S1** shows the GC trace map of the strongly water-repellent soil (T_3__S_2_) under T_3_. Straight-chain fatty acids (C_16_–C_32_) were the most abundant compounds in all soils. The distribution and relative abundance of straight-chain fatty acids beneath different plant species are shown in **Figure 4**. Fatty acids showed a strong even-over-odd preference, and the relative concentrations of fatty acids revealed differences between plant species (**Supplementary Table S5**).

#### 3.3.2 Alkanols

**Figure 5:**
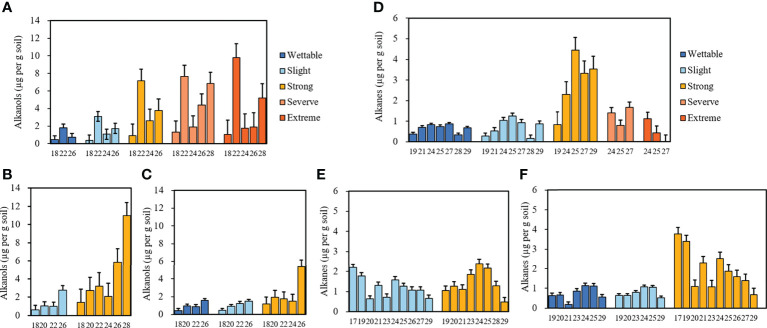
Relative concentrations of alkanols and alkanes with different carbon numbers beneath *Pinus* **(A**, **D)**, *Robinia* **(B**, **E)**, and *Hippophae* **(C, F)** (n=3).

The average concentrations of alkanols in the soils belonging to different water-repellency classes are shown in **Figures 5A** **–C**. Alkanols were the second-largest group of compounds in all soil samples, and their distribution showed a strong even-over-odd preference. The relative concentrations of alkanols increased with SWR (**Supplementary Tables S5 and S6**). Furthermore, C_18_, C_22_, and C_26_ alcohols were distributed in all soils, where C_22_ alcohol was the most abundant under T_1_. With its strong water repellency, T_2_ had the highest amount of C_28_ alcohol as compared to other soils, whereas C_26_ alcohol was the most abundant alkanol under T_3_.

#### 3.3.3 Alkanes

Alkanes were the third largest group of compounds in all soils. Their relative concentrations are shown in **Figures 5D** **–F**. They differ from alkanoic acids and alkanols, whereby alkanes (C_19_–C_29_) occurred in an odd-over-even predominance. The relative concentrations of alkanes had no relationship with SWR (**Supplementary Table S5**), the types of alkanes under T_1_ were the least, and C_25_ alkane dominated in most soils, especially in strongly water-repellent soil under T_1_, where C_24_ alkanes were also present in high proportions.

#### 3.3.4 Sterols

Apart from alkanoic acids, alkanols, and alkanes, we observed campesterol to be present in all soils. Other sterols identified here included stigmasterol and β-sitosterol. Moreover, a small amount of ergosterol and lupeol were also observed in T_1_ and T_2_, respectively. The distribution of campesterol, β-sitosterol, ergosterol, and lupeol were related to SWR (**Supplementary Table S5**). The highest sterol content occurred in strongly water-repellent soils (**Table 3**).

**Table 3:** Relative content of soil sterols beneath the different samples.

Sample plots	Treatments	Campesterol(µg·g^−1^ soil)	Stigmasterol(µg·g^−1^ soil)	β-Sitosterol(µg·g^−1^ soil)	Ergosterol(µg·g^−1^ soil)	Lupeol(µg·g^−1^ soil)
T_1_	T_1__W	2.47 ± 0.711de	0.89 ± 0.094e	5.91 ± 0.288e	1.57 ± 0.161c	—
	T_1__S_1_	2.79 ± 0.437de	2.16 ± 0.135bcde	10.02 ± 1.226d	1.8 ± 0.449c	—
	T_1__S_2_	8.15 ± 0.219a	3.54 ± 1.197bc	30.4 ± 0.528a	6 ± 0.279a	—
	T_1__S3	4.44 ± 0.208c	3.39 ± 1.378bcd	17.56 ± 0.364b	3.71 ± 0.411b	—
	T_1__E	5.11 ± 1.126bc	2.14 ± 0.897bcde	17.5 ± 2.262b	3.09 ± 0.332b	—
T_2_	T_2__S_1_	2.07 ± 0.14e	2.45 ± 0.296bcde	5.75 ± 0.716e	—	2.66 ± 0.495b
	T_2__S_2_	5.66 ± 0.838b	6.48 ± 0.828a	13.63 ± 1.112c	—	7.2 ± 0.581a
T_3_	T_3__W	1.7 ± 0.088e	1.75 ± 0.043cde	5.54 ± 0.61e	—	—
	T_3__S_1_	1.55 ± 0.075e	1.58 ± 0.042de	4.82 ± 0.218e	—	—
	T_3__S_2_	3.51 ± 0.262d	3.78 ± 0.587b	12.21 ± 0.409c	—	—

Values are means ± SE (n = 3). Different letters indicate statistically significant differences (p< 0.05) between the different samples by ANOVA. The number of soil samples is 30.

**Figure 6:**
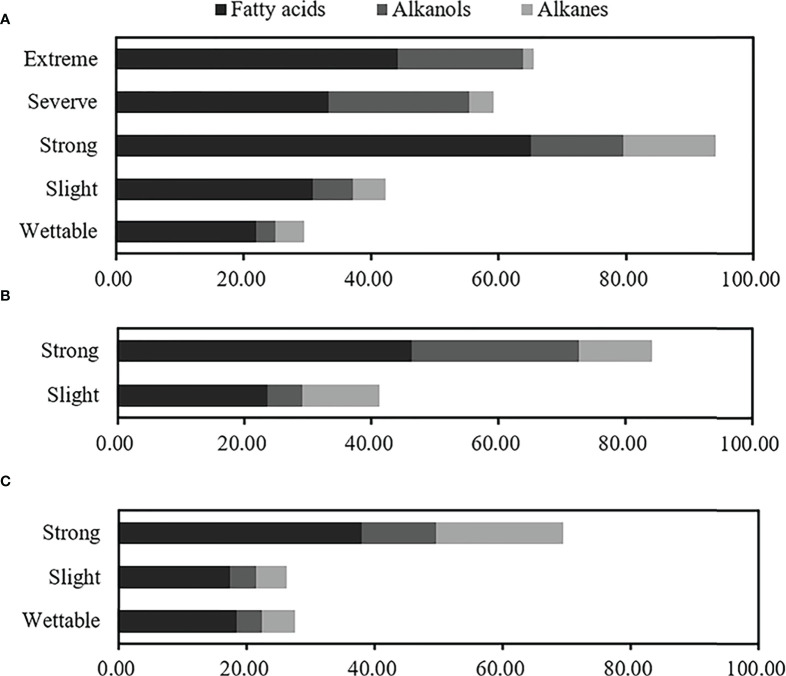
Relative concentrations of total free lipids at different water-repellency classes beneath the *Pinus* **(A)**, *Robinia* **(B)**, and *Hippophae* **(C)**.

Overall, even-numbered long-chain alkanoic acids (C_16_–C_32_) and alkanols (C_18_–C_28_), along with odd-numbered long-chain alkanes (C_19_–C_29_), were the dominant hydrophobic compounds observed in water-repellent soils. The relative concentrations of total free lipids in soils belonging to different water-repellency classes were as follows: *Pinus* (T_1_) > *Robinia* (T_2_) > *Hippophae* (T_3_). In addition, the occurrence of SWR required different free lipids in different plant species (**Supplementary Table S6**). The highest fatty acids, alkanes, and total free lipids have occurred in strongly water-repellent soil (**Figure 6**). Unlike alkanes, the content of alkanols increased with SWR and reached the highest level in severely and extremely water-repellent soil. **Figure 6** shows that even if the soil contained a higher amount of alkanol, the SWR was not stronger, e.g., the relative concentration of alkanol in strongly water-repellent soil under T_2_ was similar to that in extremely water-repellent soil under T_1_ (**Supplementary Table S6**).

### 3.4 Soil microbial community

#### 3.4.1 Soil microbial community abundance and α-diversity analysis

**Figure 7:**
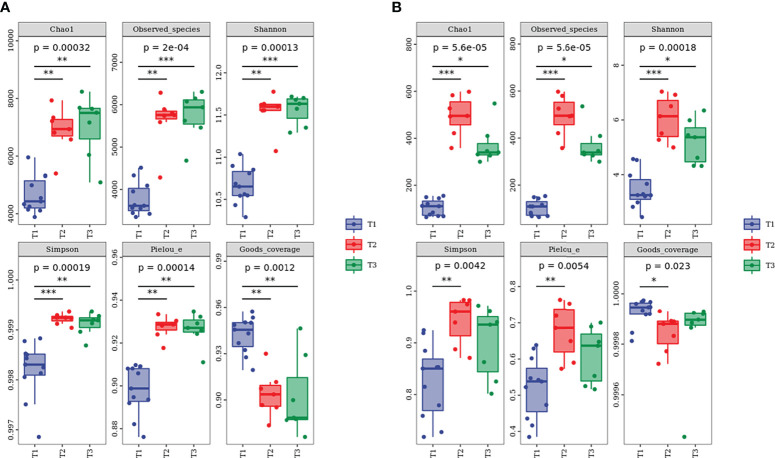
Alpha diversity index of **(A)** bacterial and **(B)** fungal communities at different water-repellency classes beneath the *Pinus* (T_1_), *Robinia* (T_2_), and *Hippophae* (T_3_). * indicates p <0.05, ** indicates p <0.01, *** indicates p< 0.001.

The 2,113,597 and 2,187,475 raw sequences for bacteria and fungi were obtained by Illumina NovaSeq PE250 high-throughput sequencing. After quality filtering, bacterial and fungal sequences per sample were normalized to 20,701 and 57,589 sequences, which were the smallest among all samples, respectively. The relatively high Good’s coverage values ranging from 0.9200 to 0.9999 indicated that microbial communities were well sampled owing to the high depth of Illumina sequencing (**Figure 7**). Furthermore, the tendency of the sparse curve of each sample to be flat (**Supplementary Figure S2**) suggested that the extent of sequencing in this study was sufficient and could accurately reflect the real information about soil microbial communities. In addition, the alpha diversity indexes of bacteria and fungi under the three species were as follows (**Figure 7**): T_2_ ≈ T_3_ > T_1_.

#### 3.4.2 Taxonomic composition and correlation analysis

**Figure 8:**
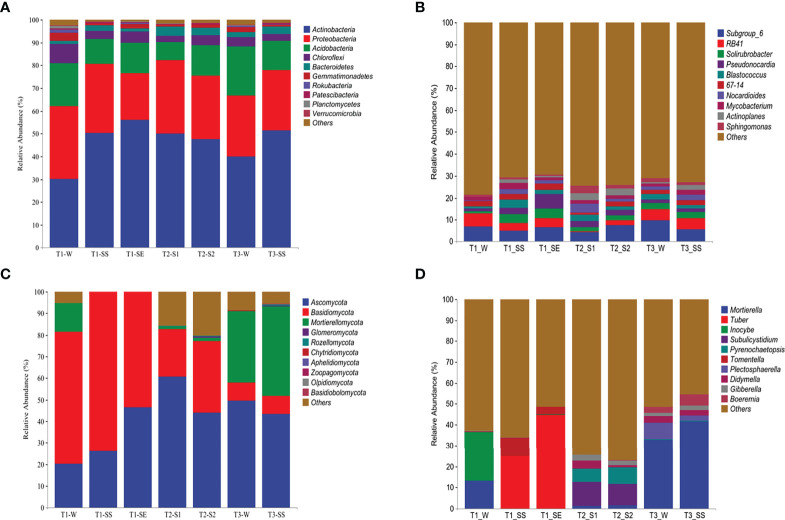
Taxonomic proportions of bacterial and fungal diversities beneath *Pinus* (T_1_), *Robinia* (T_2_), and *Hippophae* (T_3_) at phylum **(A, C)** and genus **(B, D)** levels.

Based on the classifiable sequences, the bacterial reads were mostly assigned to the same 10 phyla under T_1_, T_2_, and T_3_ in the following order: *Actinobacteria* (47.34%), *Proteobacteria* (27.66%), *Acidobacteria* (13.95%), *Chloroflexi* (4.25%), *Bacteroidetes* (2.63%), *Gemmatimonadetes* (1.54%), *Patescibacteria* (0.39%), *Rokubacteria* (0.37%), *Planctomycetes* (0.27%), and *Verrucomicrobia* (0.21%) (**Figure 8A**). Moreover, the distributions of relative abundances across the bacterial genera were also similar under T_1_, T_2_, and T_3_ (**Figure 8B**).

The dominant phyla of fungi varied greatly under T_1_, T_2_, and T_3_ (**Figure 8C**). However, *Ascomycota* was the top phylum under both T_2_ and T_3_, while the *Basidiomycota* was the dominant phylum under T_1_. At the genus level (**Figure 8D**), the most abundant fungal taxon varied by vegetation types, e. g., *Subulicystidium* and *Mortierella* were the top genera under T_2_ and T_3_, respectively. Significantly, when different classes of water repellency were considered, the dominant fungal genus changed under T_1_, e.g., *Inocybe* and *Mortierella* had higher relative abundances under T_1__W, while *Tuber* and *Tomentella* were more dominant than *Inocybe* and *Mortierella* under T_1__SS and T_1__SE.

Correlation analysis (**Supplementary Figure S3**) showed that *Actinobacteria* in dominant bacteria was positively correlated with SWR, and *Mortierellomycota* in dominant fungi was negatively correlated with SWR (*p<* 0.01).

#### 3.4.3 β-Diversity analysis

**Figure 9:**
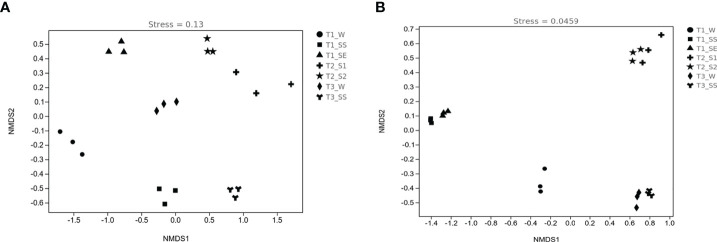
Nonmetric multidimensional scaling (NMDS) ordinations of bacterial **(A)** and fungal **(B)** diversities in soils belonging to different water-repellency classes beneath *Pinus* (T_1_), *Robinia* (T_2_), and *Hippophae* (T_3_).

The patterns of bacterial and fungal β-diversity were visualized with NMDS plots (**Figures 9A, B** **)**. The overall pattern of bacteria was differentiated into seven clusters by groups, but fungi were divided into four clusters according to the vegetation types (except T_1_), e.g., T_2__S_1_ and T_2__S_2_ as cluster 1 and T_3__W and T_3__SS as cluster 2. On the contrary, T_1__W constituted cluster 3, T_1__SS and T_1__SE formed cluster 4, which did not show the same characteristics as T_2_ and T_3_. As compared to bacteria, fungal communities were more closely gathered in the different classes of water-repellent soils.

#### 3.4.4 Linear discriminant analysis effect size analysis

**Figure 10:**
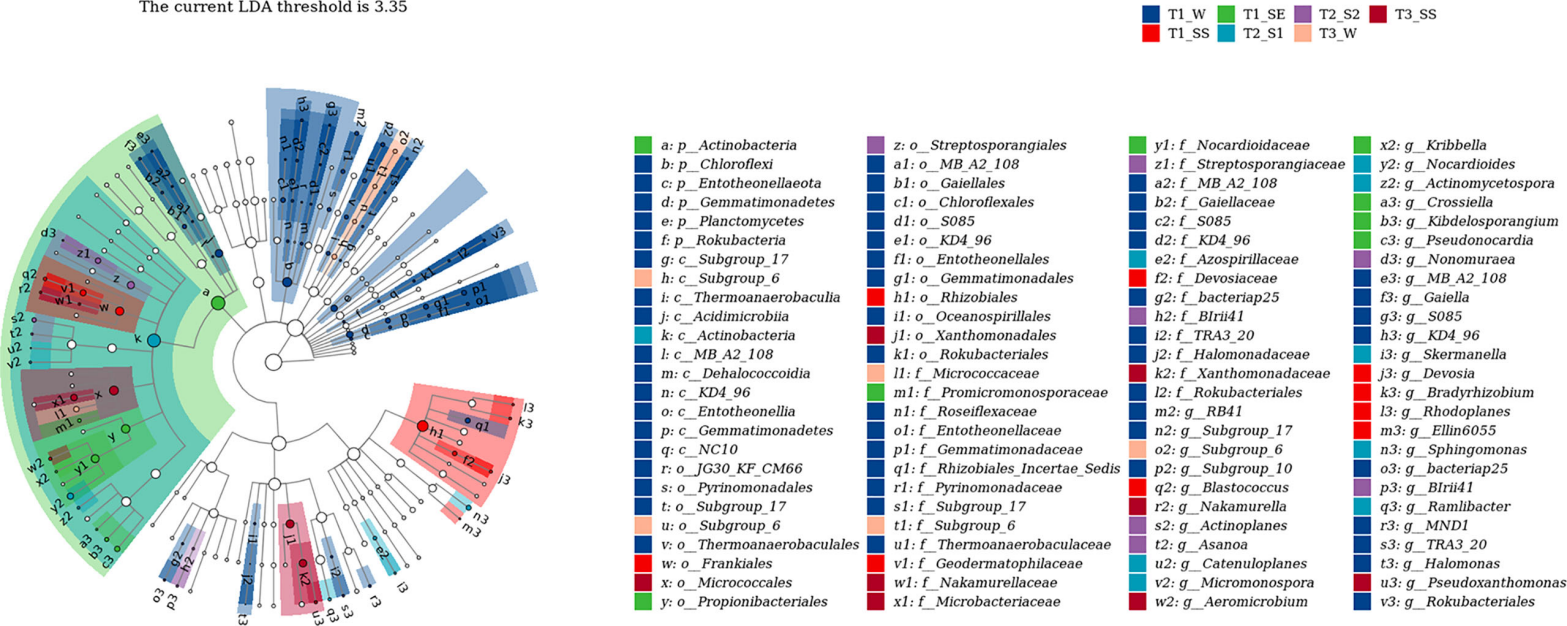
Linear discriminant analysis (LDA) effect size (LefSe) for bacteria in soils belonging to different water-repellency classes beneath *Pinus* (T_1_), *Robinia* (T_2_), and *Hippophae* (T_3_).

**Figure 11:**
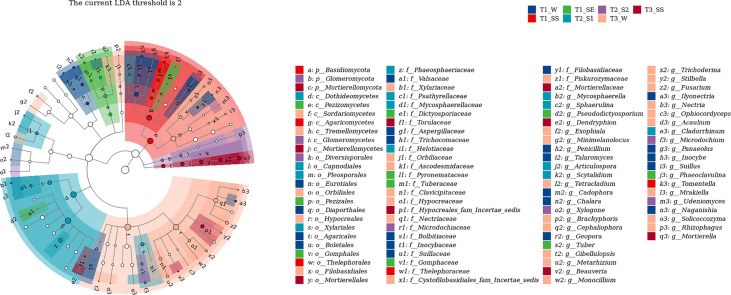
Linear discriminant analysis (LDA) effect size (LefSe) for fungi in soils belonging to different water-repellency classes beneath *Pinus* (T1), *Robinia* (T2), and *Hippophae* (T3).

We also used LEfSe to determine which taxa were most likely to explain the differences among the water-repellency classes under three vegetation types. Each ASV with an LDA value of >2 was collected, while the higher LDA values represented greater differences. A total of 36 bacterial and 42 fungal genera were obtained, respectively. The *Actinobacteria* in bacteria was significantly enriched under T_1__SE at the phylum level. Under T_1__W, T_1__SS, T_1__SE, T_2__S_1_ T_2__S_2_, T_3__W, and T_3__SS, 2, 1, 4, 4, 3, 0, and 2 genera were enriched, respectively (**Figure 10**). However, *Basidiomycota*, *Glomeromycota*, and *Mortierellomycota* were enriched with a high LDA score under T_1__SS, T_2__S_2_, and T_3__SS at the phylum level (**Figure 11**). From phylum *Basidiomycota*, 4, 1, 1, 1, 1, 4, and 0 genera were enriched under T_1__W, T_1__SS, T_1__SE, T_2__S_1_ T_2__S_2_, T_3__W, and T_3__SS, respectively.

## 4 Discussion

### 4.1 Distribution characteristics of loess SWR

A measure of vegetation is one of the most useful and basic methods to harness the typical soil and water loss area of the Loess Plateau in China. The horizontal distribution of SWR under the three common plants introduced during revegetation in a loess hilly region was in the form of patches, which was consistent with a large number of previous works in other arid and semi-arid forest ecosystems (Mataix-Solera et al., 2007), implying that SWR has a high variability (Lozano et al., 2013). SWR promoted topsoil resistance to infiltration, presenting highly non-uniform vertical wetting patterns in the soil; deep-rooted plants can take advantage of such non-uniform wetting patterns to resist drought stress (Bachmann et al., 2001; Shahidzadeh-Bonn et al., 2007: Gupta et al., 2015; Rye and Smettem, 2018). In agreement with these studies, our work suggested that under *Pinus*, the SWR of loess was more serious, and the proportion of severely to extremely water-repellent soils was nearly 50%. However, the severity of SWR that occurred in the other two common vegetations was relatively lighter, and most of the soil was slightly or strongly water repellent. Therefore, the persistence of SWR depends on the influence of vegetation species in the loess. Largely, for this reason, the soil is provided with different inputs of organic compounds. In general, evergreen trees of the same age provide more organic matter and hydrophobic compounds than deciduous plants, leading to stronger SWR. As was expected, we procured the most serious water-repellent soil samples from *Pinus*. The *in-situ* WDPT test showed that the average WDPT value of *Robinia* and *Hippophae* forestland was 65 s (**Supplementary Table S7**), indicating that SWR of loess under *Robinia* and *Hippophae* would increase surface runoff and cause soil erosion in approximately the first 65 s after rainfall. These data can be used for check-dam construction, erosion sediment yield control, and sediment transport reduction in the future. Additionally, our study further verified that most loess exhibited subcritical water repellency, which can be corroborated by a large number of works studying sandy soils. Therefore, in arid and semi-arid ecosystems, soil water repellency should be regarded as the norm rather than the exception (Smettem et al., 2021).

### 4.2 SWR and soil physicochemical factors

A negative correlation was noted between pH and SWR, i.e., pH decreased with SWR, which has been reported previously and is attributable to the formation of organic acids after SOM decomposition (Zavala et al., 2009). In this research, from wettable to strongly repellent soils, SWR had a strong correlation with SOC but a weak correlation with AN and AK, which is consistent with previous works (Harper et al., 2000; Mataix-Solera and Doerr, 2004; Doerr et al., 2005; Hermansen et al., 2019). With an increase in organic matter content, the number of hydrophobic compounds considerably increased. In turn, the soil particle surface was covered with more compounds, causing the intensification of SWR. Therefore, SOM greatly contributes to SWR. We also found different water-repellency classes among *Pinus*, *Robinia*, and *Hippophae*, despite the soils under them having similar SOM content. This inconsistency has been attributed to the fact that SWR can be controlled by the type and quality of SOM rather than by its amount (Wallis and Horne, 1992; Dekker and Ritsema, 1994; De Blas et al., 2010). In addition, there was no difference in SOC content in strong to extreme water-repellent soil under *Pinus*. Here, we conclude that the quality of SOC (i.e., hydrophobic compounds) determines the severity and persistence of SWR when the cumulative amount of SOC in loess exceeds 5.85%. In our work, GC-MS was used to measure the types and relative concentration of hydrophobic compounds, i.e., weakly polar n-alkanoic acids, n-alkanols, some sterols, and non-polar n-alkanes. The distribution of long-chain fatty acids (C_16_–C_32_) showed an even-over-odd preference. Similar to fatty acids, long-chain alkanols (C_18_–C_28_) also showed an even-over-odd advantage, but this advantage was stronger than that for alkanoic acids. In contrast, the distribution of alkanes (C_19_–C_29_) had an advantage of odd-over-even. These findings demonstrated that the main sources of fatty acids, i.e., alkanols and alkanes, were a characteristic of higher plants (Mao et al., 2014). A very small number of odd-numbered alkanoic acids and alkanols and even-numbered alkanes may come from plants (Wiesenberg et al., 2004) or microorganisms (Nierop et al., 2006). C_26_ alcohol was dominant in the majority of our samples, which typically indicated grasses (Van Bergen et al., 1997). C_29_ alkane was distributed in all samples, suggesting a predominant leaf input (Bull et al., 2000; Nierop et al., 2006; Mao et al., 2014). Hence, most of these hydrophobic substances identified in this work were generated by plants, and a very small amount was produced by microbes.

**Figure 12:**
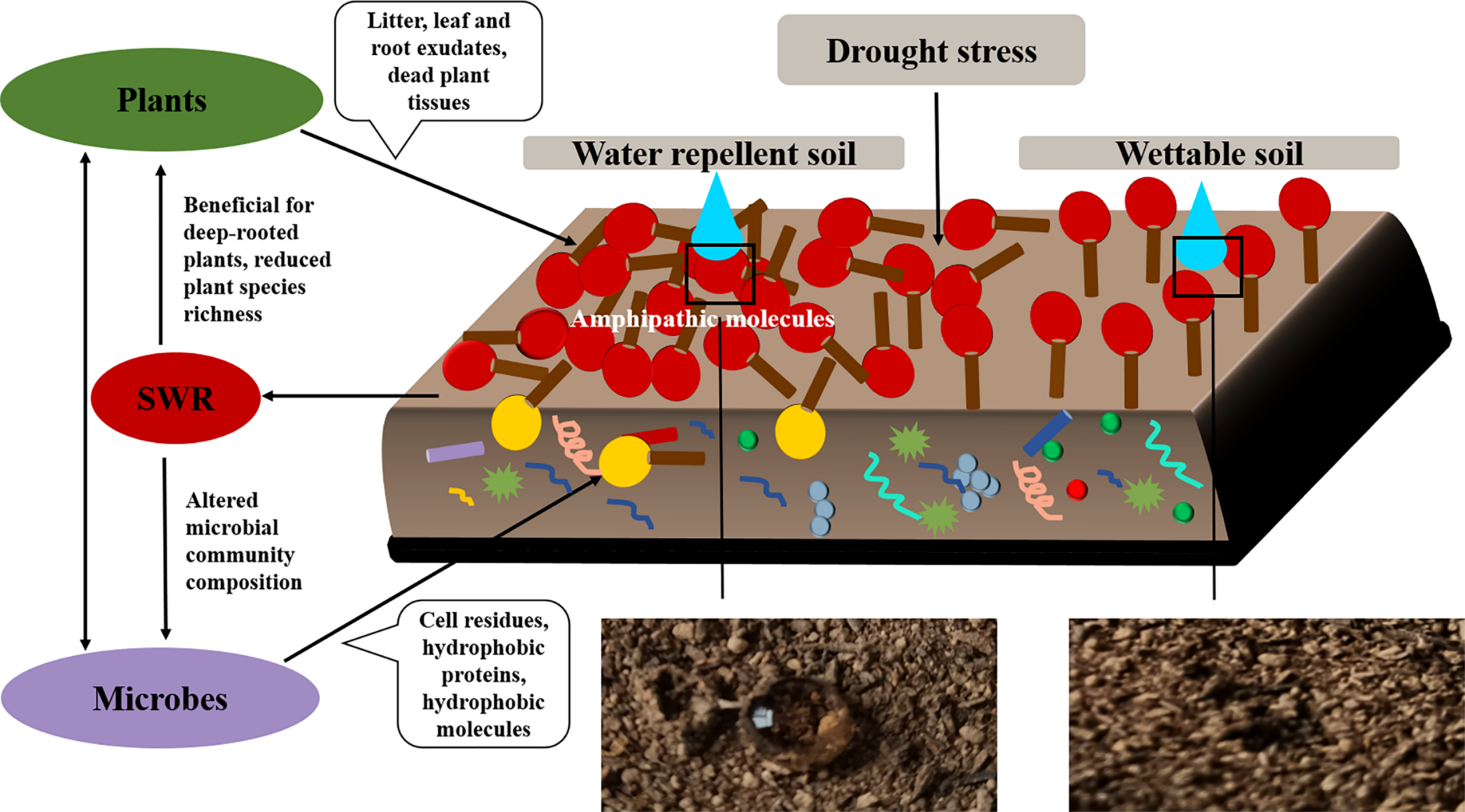
The correlation between plants, microorganisms, and SWR.

We also observed that SWR increased with fatty acids and alkanols, but alkanes had no correlation with SWR. Moreover, there were only a few kinds and smaller quantities of alkanes under *Pinus*. Therefore, we deduced that the predominant reasons for the development of SWR were the coating of soil particles by hydrophobic compounds of organic origin and the sparse existence of compounds (especially polar molecules). These findings are consistent with previous study (Doerr et al., 2000b). In addition, Horne and McIntosh (2000) reported that SWR is determined by the composition or properties of the outermost layer of organic materials, particularly amphipathic compounds, rather than the characteristics of the bulk SOM. In short, the emergence of SWR may be due to the interaction between water molecules and polar molecules, where the polar molecules are composed of a hydrophilic group (head) and a hydrophobic chain (tail; **Figure 12**). When the cohesion between water molecules is greater than the force between water and the soil surface, the soil surface shows water repellence. When the hydrophobic coating encounters water droplets, the force between them changes, reorienting and organizing the amphiphilic molecules. Once the attraction between water molecules and the soil surface is greater than the cohesion among the water molecules, the hydrophilic heads of polar amphiphilic molecules face outward, making the soil wettable (Doerr et al., 2000b; Zavala et al., 2009; Kaiser et al., 2015; Mao et al., 2018). Similarly, campesterol, stigmasterol, and β-sitosterol were also observed in all soils. Inconsistent with research by Mao et al. (2015), the distribution of campesterol, β-sitosterol, ergosterol, and lupeol in all soils was related to SWR. This discrepancy could be because they used sandy soil from different vegetation types and geographical locations, which may have different effects on SWR. In this research, however, the loess we used was all from the same site.

In summary, the distribution of free lipids in our soil largely originated from plant leaf wax, and the contribution of free lipids to SWR was observed, which agreed with some previous studies (Van Bergen et al., 1997; DeBano, 2000; Franco et al., 2000; Mao et al., 2014). However, some other studies did not find a correlation between SWR and lipids or any lipid parts (Horne and McIntosh, 2000; Mao et al., 2014). We also found similar alkanol abundance between *Pinus* and *Robinia*, but they belonged to different water-repellency classes. Therefore, polar wax may be a necessary condition that contributes to SWR, but it is not sufficient on its own. In our work, leaf waxes were the main free lipid extracts under the three vegetation types, but the ester binding lipids (e.g., cutin and suberin) from the leaves and roots were not considered. These stable carbon sources in soil may be degraded monomers produced from microbial hydrolysis (Naafs et al., 2004), e.g., fatty acids, alkanols, ω-hydroxy fatty acids, and α, ω-dicarboxylic acid, which can directly affect SWR (Mao et al., 2014). Thus, we will focus on the distribution of hydrophobic compounds from cutin and suberin in loess and their relationship with SWR in subsequent studies.

### 4.3 SWR and soil microbes

In addition to specific substances such as long-chain fatty acids, microbes themselves—and their cell residues (which always will be present during growth and decay)—contribute to SOM formation and, thus, have an important effect on the wettability of soil (Miltner et al., 2012; Schurig et al., 2012; Achtenhagen et al., 2015). In this study, we found a small amount of ergosterol under *Pinus*, and the abundance of *Basidiomycota* under *Pinus* was higher than that under *Robinia* and *Hippophae*. Bayry et al. (2012) suggested that arbuscular mycorrhizae (AM) can produce a glycoprotein called GRSP, whose presence is associated with hydrophobicity (Young et al., 2012). Actinomycetes and basidiomycetes can generate amphiphilic hydrophobic proteins forming fairy rings that induce SWR (Unestam, 1991; Wessels, 1997; Wessels, 2000; York and Canaway, 2000; Epstein et al., 2010; Bayry et al., 2012; Spohn and Rillig, 2012). We speculate that SWR is likely linked to fungi under *Pinus*, which is in line with the results of Lozano et al. (2013). However, the inference that fungi are responsible for SWR may need further validation through traditional microbial isolation and identification methods.

In this work, we found that the factors affecting SWR are not limited to the accumulation of SOM but also include the blocked diversity of microbial community causing accumulation of hydrophobic substances. The changes in SWR may be a significant source of stress for microbial communities (Denef et al., 2001). It can also alter community composition and growth of microorganisms by selecting soil microbes that can adapt to rapid changes in water content (Fierer et al., 2003). Under extreme climatic conditions, the effects of SWR on plant and soil microflora are amplified as a form of disturbance in water infiltration. In the case of limited water supply, both plant moisture and microbial activity are limited by SWR, which affects SOM decomposition (Mao et al., 2018). Through high-throughput sequencing, we found that α-diversity index under *Pinus*, such as Chao1 index, observed species index, Simpson index, Shannon–Wiener index, and Pielou’s evenness index, were lower than those under *Robinia* and *Hippophae*, implying that some hydrophobic substances could not be decomposed in time due to the lack of soil microbial diversity under *Pinus*. β-Diversity analysis showed the variation in the fungal community under *Pinus* to be larger than that under *Robinia* and *Hippophae*. Conversely, the water-repellent and wettable soil under *Pinus* did not gather in one cluster but formed two different clusters, which may be attributable to the greater variation in the microbial community under *Pinus* and may lead to severe and extreme water repellency (Seaton et al., 2019). The indicator species mainly belonged to *Actinobacteria* in bacteria and *Basidiomycota* in fungi at the phylum level, which was further supported by LEfSe analysis. Distinctly, SWR was more closely related to soil microbes than previously understood, manifesting the significance of ecology in altering hydrological processes through feedback. Moreover, this feedback mechanism would contribute to soil and water conservation. Consequently, SWR under *Pinus* appeared to be the most influenced by fungi and actinomycetes, when the topsoil was close to being air-dry.

## 5 Conclusion

In this study, SWR recorded under three dominant vegetations in the loess hilly region was generally distributed in patches. We observed severely or extremely water-repellent soils only under *Pinus*, while the soils under *Robinia* and *Hippophae* showed slight or strong water repellence. Therefore, it is necessary to introduce reasonable vegetation according to the water-repellency properties of different vegetation types in the soil to maximize water conservation. The major factor affecting SWR is no longer understood to be the quantity of SOC, but a small number of polar waxes are prominent drivers of SWR as well. In addition, the α-diversity index under *Pinus* was significantly lower than those under *Robinia* and *Hippophae*. NMDS showed the variation in the fungal community under *Pinus* to be larger than that under *Robinia* and *Hippophae*. The indicator species mainly belonged to *Actinobacteria* in bacteria and *Basidiomycota* in fungi at the phylum level, which was further supported by LEfSe. Moreover, GC-MS identified a small amount of ergosterol under *Pinus*. Here, we showed that SWR recorded under *Pinus* was most influenced by fungi and actinomycetes, when the topsoil was close to air-drying. However, SWR is a complex property caused by numerous interconnected soil parameters. In our study, we have tried to explain which factors are the most relevant in the development of SWR in loess. According to our results, extractable polar waxes are the most relevant factor under the given conditions. Therefore, it is essential to conduct further studies on loess with different vegetations or land-use types. Overall, the results of this study highlight the significant impacts of polar waxes on loess wetting properties. Furthermore, fungi and actinomycetes can lead to severe to extreme SWR under *Pinus*.

## Data availability statement

The datasets presented in this study can be found in online repositories. The names of the repository/repositories and accession number(s) can be found below: https://www.ncbi.nlm.nih.gov/bioproject/PRJNA823826; https://www.ncbi.nlm.nih.gov/bioproject/PRJNA820436.

## Author contributions

XC and XX designed the study. XC, WW, PG, YQ, QZ, XR, YX, and ML performed fieldwork and did experiments with the help of SL and LL. XC carried out statistical and bioinformatics analyses, prepared figures and tables, interpreted the results, and wrote the manuscript with the help of XX. XX reviewed and edited the manuscript. All authors contributed to the article and approved the final version.

## Funding

This research was supported by the National Natural Science Foundation of China (Grant No. 41977426).

## Acknowledgments

We would like to thank the staff of Changwu Agro-Ecological Experiment Station for their assistance in field investigations and sample collections. Sequencing service was provided by Shanghai Personal Biotechnology Co., Ltd., China. The RDA analysis was performed by the Genescloud tools (https://www.genescloud.cn), a free online platform for data analysis.

## Conflict of interest

The authors declare that the research was conducted in the absence of any commercial or financial relationships that could be construed as a potential conflict of interest.

The reviewer YT declared a shared affiliation with the author XC, XX, WW, SL, PG, YQ, QZ, XR, YX, and ML to the handling editor at the time of review

## Publisher’s note

All claims expressed in this article are solely those of the authors and do not necessarily represent those of their affiliated organizations, or those of the publisher, the editors and the reviewers. Any product that may be evaluated in this article, or claim that may be made by its manufacturer, is not guaranteed or endorsed by the publisher.
