# Supplementary material for: Physicochemical and biological factors determining the patchy distribution of soil water repellency among species of dominant vegetation in loess hilly region of China
Source: Front Plant Sci. 2022 Oct 6;13:908035. doi: 10.3389/fpls.2022.908035 (PMC9582861; doi:10.3389/fpls.2022.908035)
Supplement: Supplementary file 1 [file DataSheet_1.docx]

Supplementary Material

# Supplementary Figures


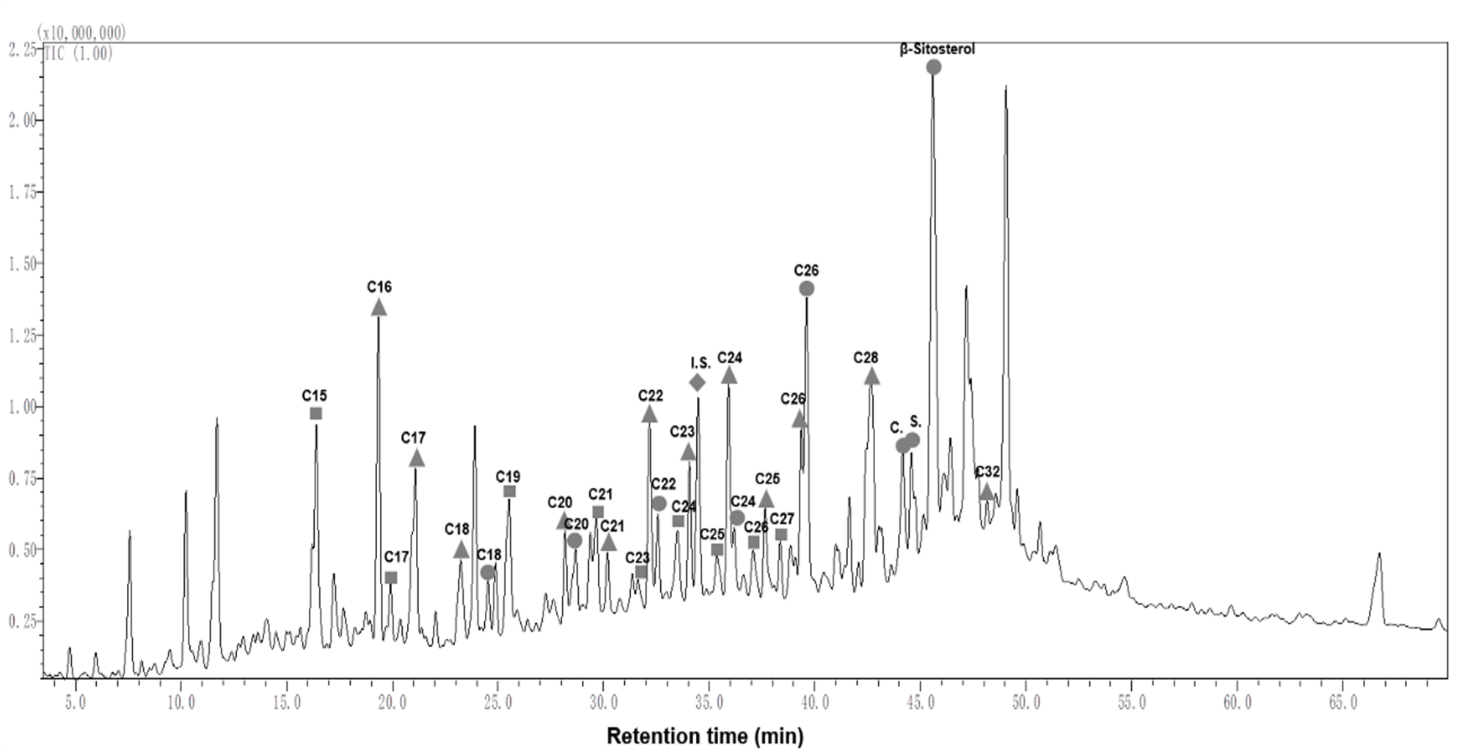


**Supplementary Figure S1.** Gas chromatograms of free lipids from T_3_-S_2_ (beneath *Hippophae*, strong water-repellent sample). Legend: ▲: n-fatty acids; ●: n-alcohol; ■: n-alkanes; ◆: I.S.- internal standard; C. - Campesterol; S.- Stigmasterol; Cn indicates chain length.


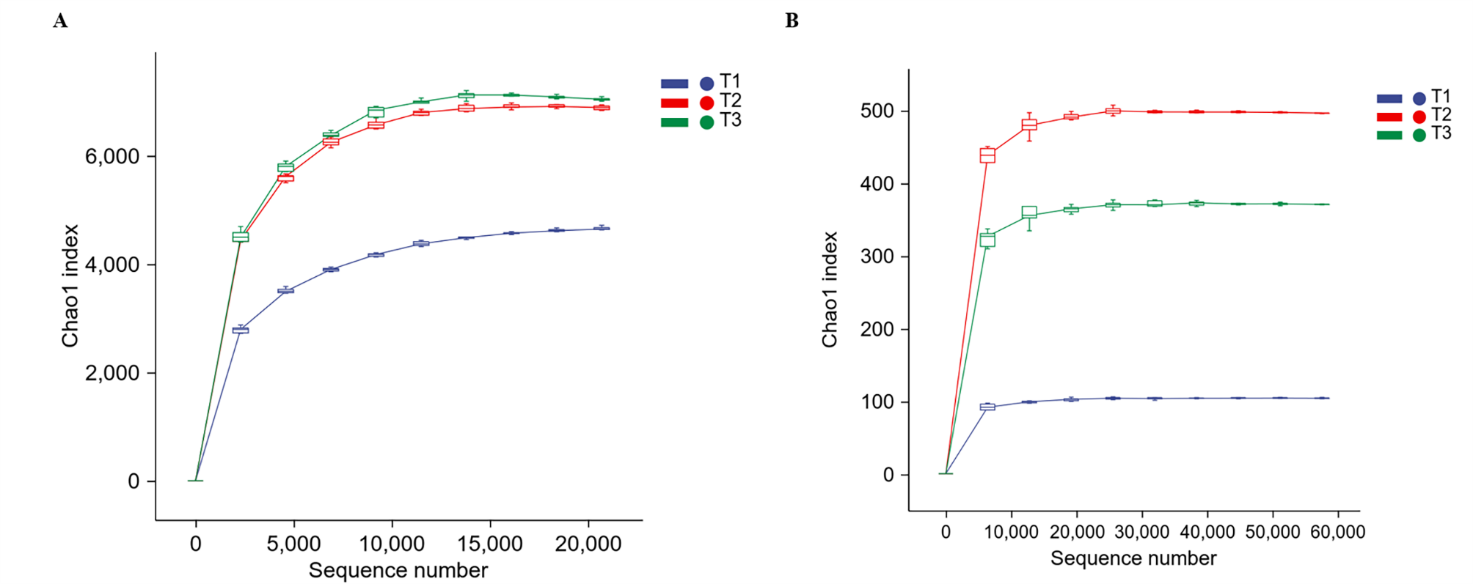


**Supplementary Figure S2.** Rare faction curves for (A)bacterial and (B)fungal DNA samples beneath the *Pinus* (T_1_), *Robinia* (T_2_), *Hippophae* (T_3_).


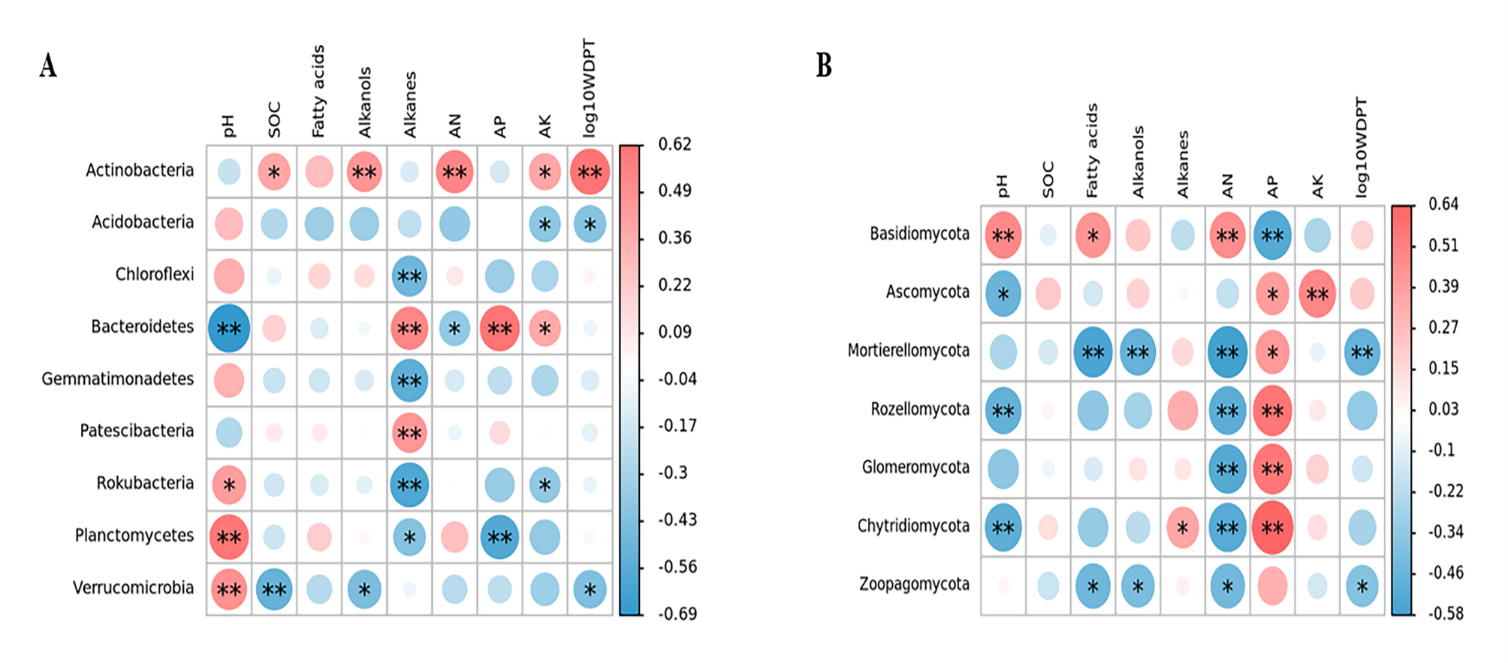


**Supplementary Figure S3.** Correlation analysis of dominant bacterial (A) or fungal (B) communities with SWR.

# Supplementary Tables

**Supplementary Table S1.** Soil texture of each water-repellent class under different plant species.

| Vegetation types | Treatments | Clay <0.002(%) | Silt  0.002~0.02(%) | Sand 0.02~2(%) |  |
| --- | --- | --- | --- | --- | --- |
|  |  |  |  |  |  |
| T_1_ | T_1__W | 24.46 | 37.69 | 37.85 |  |
|  | T_1__S_1_ | 22.99 | 37.11 | 39.90 |  |
|  | T_1__S_2_ | 21.65 | 39.86 | 38.49 |  |
|  | T_1__S3 | 26.65 | 40.98 | 32.37 |  |
|  | T_1__E | 27.21 | 44.83 | 27.96 |  |
| T_2_ | T_2__S_1_ | 13.92 | 27.26 | 58.82 |  |
|  | T_2__S_2_ | 12.28 | 26.08 | 61.64 |  |
| T_3_ | T_3__W | 17.68 | 29.13 | 53.19 |  |
|  | T_3__S_1_ | 14.54 | 26.95 | 58.51 |  |
|  | T_3__S_2_ | 12.42 | 26.01 | 61.57 |  |

**Supplementary Table S2.** Relative frequency of each water-repellent class under different plant species.

| Vegetation types | Wettable | Slight | Strong | Severve | Extreme |
| --- | --- | --- | --- | --- | --- |
| Hippophae | 23.33% | 49.33% | 27.34% | 0 | 0 |
| Robinia | 0 | 44.67% | 55.33% | 0 | 0 |
| Pinus | 5.34% | 15.33% | 30% | 13.33% | 36% |

**Supplementary Table S3.** pH of each water-repellent class under different plant species.

| Vegetation types | Treatments | pH |  |
| --- | --- | --- | --- |
|  |  |  |  |
| T_1_ | T_1__W | 7.87a |  |
|  | T_1__S_1_ | 7.77b |  |
|  | T_1__S_2_ | 7.71b |  |
|  | T_1__S3 | 7.62c |  |
|  | T_1__E | 7.54d |  |
| T_2_ | T_2__S_1_ | 7.46e |  |
|  | T_2__S_2_ | 7.35f |  |
| T_3_ | T_3__W | 7.78b |  |
|  | T_3__S_1_ | 7.55d |  |
|  | T_3__S_2_ | 7.38f |  |

Values are means ±SE (n=3). Different letters show the statistically significant differences (*p* <0.05) between the different samples by ANOVA. The number of soil samples is thirty. The same below.

**Supplementary Table S4.** SOC content of each water-repellent classes under different plant species.

| Vegetation types | Treatments | SOC (%) |  |
| --- | --- | --- | --- |
|  |  |  |  |
| T_1_ | T_1__W | 1.82d |  |
|  | T_1__S_1_ | 4.00bc |  |
|  | T_1__S_2_ | 5.85 a |  |
|  | T_1__S3 | 6.08 a |  |
|  | T_1__E | 6.95 a |  |
| T_2_ | T_2__S_1_ | 3.52 bc |  |
|  | T_2__S_2_ | 6.48 a |  |
| T_3_ | T_3__W | 3.06 c |  |
|  | T_3__S_1_ | 4.57 b |  |
|  | T_3__S_2_ | 7.01 a |  |

**Supplementary Table S5.** Correlation coefficient between soil physicochemical parameters and SWR .

| Parameters | r | *p* |
| --- | --- | --- |
| AN | 0.671 | 0.000 |
| AP | -0.054 | 0.000 |
| AK | 0.584 | 0.779 |
| Fatty acids | 0.628 | 0.001 |
| Alkanols | 0.741 | 0.000 |
| Alkanes | 0.044 | 0.816 |
| Campesterol | 0.638 | 0.000 |
| Stigmasterol | 0.393 | 0.032 |
| β-sitosterol | 0.685 | 0.000 |
| Ergosterol | 0.544 | 0.036 |
| Lupeol | 0.948 | 0.004 |

**Supplementary Table S6.** Fatty acids, alkanols, and alkanes content of each water-repellent class under different plant species.

| Vegetation types | Treatments | Fatty acids (µg·g^-1^ soil) | Alkanols  (µg·g^-1^ soil) | Alkanes (µg·g^-1^ soil) | Free lipids  (µg·g^-1^ soil) |  |
| --- | --- | --- | --- | --- | --- | --- |
|  |  |  |  |  |  |  |
| T_1_ | T_1__W | 21.91d | 3.02d | 4.52c | 29.45cd |  |
|  | T_1__S_1_ | 30.83c | 6.32d | 5.06c | 42.21c |  |
|  | T_1__S_2_ | 65.13a | 14.46bc | 14.45b | 94.04a |  |
|  | T_1__S3 | 33.25c | 22.08a | 3.87c | 59.20b |  |
|  | T_1__E | 44.23b | 19.68ab | 1.55c | 65.46b |  |
| T_2_ | T_2__S_1_ | 23.58d | 5.44d | 12.26b | 41.29c |  |
|  | T_2__S_2_ | 46.36b | 26.28a | 11.56b | 84.20a |  |
| T_3_ | T_3__W | 18.45d | 3.86d | 5.22c | 27.53d |  |
|  | T_3__S_1_ | 17.37d | 4.10d | 4.77c | 26.23d |  |
|  | T_3__S_2_ | 37.89c | 11.73cd | 19.78a | 69.40b |  |

**Supplementary Table S7.** WDPT values under different vegetation beneath the *Pinus* (T_1_), *Robinia* (T_2_), *Hippophae* (T_3_).

| Sampling plots | WDPT (s), n=150 | | | | | | | | | |
| --- | --- | --- | --- | --- | --- | --- | --- | --- | --- | --- |
| T_1__1 | 204.67 | 3.33 | 17.33 | 3.00 | 475.67 | 470.33 | 168.00 | 2487.33 | 80.33 | 2454.33 |
|  | 60.67 | 308.33 | 206.33 | 421.00 | 1612.67 | 809.00 | 1244.33 | 1007.67 | 364.67 | 4859.00 |
|  | 44.67 | 25.33 | 15.00 | 68.67 | 646.67 | 441.67 | 1235.67 | >3600 | >3600 | 4953.33 |
|  | 36.00 | 94.33 | 6.67 | 3.00 | >3600 | >3600 | >3600 | >3600 | >3600 | >3600 |
|  | 31.00 | 36.67 | 33.67 | 29.67 | >3600 | >3600 | >3600 | >3600 | >3600 | 913.33 |
|  |  |  |  |  |  |  |  |  |  |  |
| T_1__2 | 114.67 | 525.67 | 324.00 | 25.00 | 367.67 | 660.33 | 259.33 | 21.33 | 22.33 | 109.00 |
|  | >3600 | 518.00 | 178.33 | 1098.33 | 364.67 | 905.67 | 659.00 | >3600 | 627.67 | 58.67 |
|  | 86.00 | >3600 | >3600 | 211.00 | >3600 | 0.33 | >3600 | >3600 | >3600 | >3600 |
|  | 0.33 | >3600 | >3600 | 499.67 | >3600 | >3600 | >3600 | 34.67 | 63.33 | >3600 |
|  | 120.33 | >3600 | >3600 | 372.67 | 305.33 | >3600 | >3600 | 266.33 | >3600 | >3600 |
|  |  |  |  |  |  |  |  |  |  |  |
| T_1__3 | 8.67 | 93.67 | 109.67 | 26.00 | >3600 | >3600 | >3600 | >3600 | >3600 | >3600 |
|  | 1.33 | 7.67 | 2.67 | 45.33 | 121.33 | >3600 | >3600 | >3600 | >3600 | >3600 |
|  | 4.33 | 28.00 | 12.33 | 17.00 | 122.00 | 590.33 | >3600 | >3600 | 726.33 | >3600 |
|  | 317.33 | 356.33 | 82.00 | 14.67 | >3600 | 1059.67 | 486.33 | >3600 | >3600 | 146.00 |
|  | 117.33 | 336.67 | 1119.33 | 504.33 | 1216.33 | 1116.33 | 252.00 | >3600 | 1178.00 | 138.00 |
|  |  |  |  |  |  |  |  |  |  |  |
| T_2__1 | 9.00 | 266.00 | 134.33 | 100.33 | 41.33 | 57.33 | 24.00 | 107.00 | 149.00 | 83.33 |
|  | 11.33 | 46.33 | 35.33 | 77.33 | 50.33 | 20.00 | 25.67 | 131.00 | 183.67 | 21.33 |
|  | 31.33 | 119.00 | 72.67 | 118.00 | 74.67 | 29.00 | 15.33 | 38.33 | 49.33 | 66.33 |
|  | 11.67 | 262.33 | 61.00 | 34.33 | 90.67 | 12.67 | 36.00 | 57.33 | 92.67 | 134.00 |
|  | 66.33 | 12.00 | 175.00 | 43.33 | 35.33 | 23.33 | 27.33 | 31.00 | 36.67 | 39.33 |
|  |  |  |  |  |  |  |  |  |  |  |
| T_2__2 | 75.00 | 44.67 | 62.33 | 186.00 | 36.33 | 209.00 | 95.33 | 128.00 | 136.00 | 81.67 |
|  | 130.67 | 115.67 | 135.67 | 169.00 | 74.33 | 53.00 | 66.00 | 97.00 | 184.00 | 51.33 |
|  | 61.67 | 161.67 | 57.00 | 182.00 | 168.33 | 52.00 | 143.33 | 242.33 | 164.00 | 49.33 |
|  | 80.33 | 18.00 | 53.67 | 99.00 | 80.00 | 63.00 | 45.00 | 46.33 | 47.67 | 15.00 |
|  | 75.33 | 85.00 | 94.67 | 111.33 | 36.67 | 35.33 | 53.67 | 186.00 | 20.33 | 67.00 |
|  |  |  |  |  |  |  |  |  |  |  |
| T_2__3 | 123.67 | 128.67 | 42.33 | 46.67 | 119.33 | 286.00 | 167.33 | 82.00 | 26.00 | 29.67 |
|  | 90.33 | 67.00 | 111.00 | 55.33 | 86.33 | 90.00 | 80.00 | 48.33 | 40.33 | 56.00 |
|  | 44.00 | 9.67 | 70.33 | 83.00 | 95.00 | 66.33 | 98.67 | 101.00 | 42.33 | 65.00 |
|  | 21.33 | 102.00 | 56.67 | 126.67 | 77.67 | 46.33 | 96.33 | 143.67 | 65.33 | 46.67 |
|  | 33.33 | 69.33 | 55.67 | 26.67 | 58.33 | 72.33 | 32.33 | 99.00 | 125.67 | 7.67 |
|  |  |  |  |  |  |  |  |  |  |  |
| T_2__1 | 44.33 | 38.33 | 4.33 | 59.33 | 36.33 | 42.67 | 46.33 | 24.00 | 335.67 | 10.33 |
|  | 49.67 | 18.00 | 5.00 | 37.00 | 59.00 | 28.67 | 3.33 | 21.33 | 235.33 | 9.00 |
|  | 94.33 | 10.00 | 8.00 | 1.67 | 74.33 | 124.67 | 44.00 | 97.33 | 1.67 | 2.00 |
|  | 63.00 | 63.00 | 1.33 | 17.00 | 32.00 | 108.00 | 105.67 | 20.33 | 166.33 | 3.67 |
|  | 23.33 | 16.67 | 17.33 | 25.33 | 91.33 | 90.33 | 0.67 | 142.67 | 18.33 | 228.33 |
|  |  |  |  |  |  |  |  |  |  |  |
| T_2__2 | 20.67 | 204.67 | 64.67 | 71.67 | 18.67 | 58.33 | 5.67 | 2.00 | 5.33 | 2.33 |
|  | 7.00 | 51.67 | 152.33 | 9.33 | 22.00 | 145.00 | 0.67 | 0.67 | 0.67 | 2.33 |
|  | 174.00 | 158.33 | 7.67 | 17.33 | 139.67 | 63.00 | 4.00 | 1.33 | 5.00 | 7.67 |
|  | 419.67 | 77.67 | 62.33 | 24.33 | 56.33 | 25.67 | 87.67 | 52.00 | 20.00 | 37.33 |
|  | 16.33 | 213.67 | 140.67 | 46.33 | 5.33 | 47.33 | 76.67 | 13.00 | 65.00 | 113.67 |
|  |  |  |  |  |  |  |  |  |  |  |
| T_2__3 | 2.33 | 106.33 | 326.33 | 115.33 | 50.33 | 35.00 | 48.33 | 51.33 | 4.67 | 11.67 |
|  | 50.67 | 59.67 | 21.00 | 82.33 | 72.00 | 68.33 | 25.67 | 43.33 | 2.33 | 4.00 |
|  | 28.67 | 54.00 | 20.67 | 58.67 | 63.67 | 17.00 | 4.67 | 26.67 | 3.33 | 0.33 |
|  | 36.33 | 18.67 | 12.67 | 122.33 | 36.00 | 13.67 | 34.00 | 4.00 | 0.67 | 5.00 |
|  | 46.00 | 6.33 | 26.67 | 22.67 | 0.67 | 16.67 | 2.00 | 5.33 | 1.67 | 1.33 |

Mean value of WDPT tests is based on the average WDPT of 150 water individual droplets (i.e. n = 150)
